# Meclofenamic Acid Reduces Reactive Oxygen Species Accumulation and Apoptosis, Inhibits Excessive Autophagy, and Protects Hair Cell-Like HEI-OC1 Cells From Cisplatin-Induced Damage

**DOI:** 10.3389/fncel.2018.00139

**Published:** 2018-05-23

**Authors:** He Li, Yongdong Song, Zuhong He, Xiaoyun Chen, Xianmin Wu, Xiaofei Li, Xiaohui Bai, Wenwen Liu, Boqin Li, Shanshan Wang, Yuechen Han, Lei Xu, Daogong Zhang, Jianfeng Li, Renjie Chai, Haibo Wang, Zhaomin Fan

**Affiliations:** ^1^Department of Otolaryngology-Head and Neck Surgery, Shandong Provincial Hospital Affiliated to Shandong University, Jinan, China; ^2^Department of Otolaryngology-Head and Neck Surgery, First Affiliated Hospital of Wenzhou Medical University, Wenzhou, China; ^3^Shandong Provincial Key Laboratory of Otology, Jinan, China; ^4^Department of Otorhinolaryngology, Union Hospital, Tongji Medical College, Huazhong University of Science and Technology, Wuhan, China; ^5^Institute of Eye and ENT, Shandong Provincial Hospital Affiliated to Shandong University, Jinan, China; ^6^Shandong Analysis and Test Center, Jinan, China; ^7^Co-innovation Center of Neuroregeneration, Nantong University, Nantong, China; ^8^Key Laboratory for Developmental Genes and Human Disease, Ministry of Education, Institute of Life Sciences, Southeast University, Nanjing, China; ^9^Jiangsu Province Hi-Tech Key Laboratory for Bio-Medical Research, Southeast University, Nanjing, China

**Keywords:** meclofenamic acid, HEI-OC1 cells, reactive oxygen species, autophagy, cisplatin

## Abstract

Hearing loss is the most common sensory disorder in humans, and a significant number of cases is due to the ototoxicity of drugs such as cisplatin that cause hair cell (HC) damage. Thus, there is great interest in finding agents and mechanisms that protect HCs from ototoxic drug damage. It has been proposed that epigenetic modifications are related to inner ear development and play a significant role in HC protection and HC regeneration; however, whether the m^6^A modification and the ethyl ester form of meclofenamic acid (MA2), which is a highly selective inhibitor of FTO (fatmass and obesity-associated enzyme, one of the primary human demethylases), can affect the process of HC apoptosis induced by ototoxic drugs remains largely unexplored. In this study, we took advantage of the HEI-OC1 cell line, which is a cochlear HC-like cell line, to investigate the role of epigenetic modifications in cisplatin-induced cell death. We found that cisplatin injury caused reactive oxygen species accumulation and increased apoptosis in HEI-OC1 cells, and the cisplatin injury was reduced by co-treatment with MA2 compared to the cisplatin-only group. Further investigation showed that MA2 attenuated cisplatin-induced oxidative stress and apoptosis in HEI-OC1 cells. We next found that the cisplatin-induced upregulation of autophagy was significantly inhibited after MA2 treatment, indicating that MA2 inhibited the cisplatin-induced excessive autophagy. Our findings show that MA2 has a protective effect and improves the viability of HEI-OC1 cells after cisplatin treatment, and they provide new insights into potential therapeutic targets for the amelioration of cisplatin-induced ototoxicity.

## Introduction

Hearing loss is the most prevalent sensorial deficit in the general population, and it is caused by different etiologies such as congenital morphogenetic defects, aging, exposure to intense noise, ototoxic medications, and genetic disorders (Fujimoto et al., 2017; Mammano and Bortolozzi, 2017). Ototoxic drugs such as loop diuretics, aminoglycosides, and chemotherapeutics can lead to language and speech disabilities in young children due to hearing loss (Berg et al., 1999; Qaddoumi et al., 2012). Cisplatin is one of the main agents in the majority of chemotherapy protocols for the treatment of human solid tumors (Youn et al., 2015); however, cisplatin damages the stereocilia, mitochondria, and nuclei of hair cells (HCs), and this triggers apoptosis and subsequent hearing loss (Rybak et al., 2007). Thus, the ototoxicity induced by cisplatin restricts its utility and therapeutic profile in both children and adult patients (Rybak, 2007; Langer et al., 2013; Olgun, 2013), and finding an effective way to prevent cisplatin-induced HC loss remains an unmet medical need.

Epigenetic modifications are important for many biological and pathogenic processes such as transcriptional regulation, cell differentiation, survival, and cell death as well as physiological and pathological processes involved in hearing (Provenzano and Domann, 2007; Reik, 2007; Papp and Plath, 2011). Studies have also shown that the misregulation of specific epigenetic modifications can lead to developmental disorders and cancer (Layman and Zuo, 2017). Although epigenetic research has become a major focus in many tissues and biological systems, little is understood about epigenetic mechanisms in hearing pathologies or in the process of ototoxic drug-induced hearing loss. In the auditory field, there are myriad benefits to discovering epigenetic mechanisms and developing epigenetically targeted pharmaceuticals in the prevention and treatment of ototoxic hearing loss.

The m^6^A RNA modification is a dynamic process, and its dysregulation contributes to obesity, brain development abnormalities, and other diseases through dysregulation of RNA metabolism, including RNA stability, splicing, translation, transport, and localization (Klungland and Dahl, 2014; Geula et al., 2015). FTO (fat mass and obesity-associated protein) and ALKBH5^8^ are two m^6^A demethylases that control mRNA metabolism by catalyzing the demethylation of m^6^A (Wang et al., 2014). The non-steroidal, anti-inflammatory drug meclofenamic acid (MA) is a highly selective inhibitor of FTO that competes with FTO binding for m^6^A-containing nucleic acids (Huang et al., 2015). Previous studies showed that the ethyl ester form of MA (MA2) can be used to detect the levels of m^6^A modifications in mRNA (Huang et al., 2015). In this study, we investigated the role of m^6^A modification in the process of cisplatin-induced cell death after MA2 treatment in HEI-OC1 cells.

Previous studies have shown that the accumulation of reactive oxygen species (ROS) is an important mechanism behind the toxicity of cisplatin in HCs (Choi et al., 2014). Autophagy is an important cell survival process that recycles unnecessary or dysfunctional cellular components in response to stress (Cecconi and Levine, 2008; Kroemer and Levine, 2008; He et al., 2017). However, excessive autophagy activation can also promote cell death and pathological changes (Clarke, 1990; Yu and Lenardo, 2004; Czaja et al., 2013; Ryter et al., 2014).

In this study, we explored the function of MA2 in the process of cisplatin-induced injury in the HEI-OC1 cell line. We found that both ROS accumulation and apoptosis were reduced after MA2 treatment and that MA2 inhibited the cisplatin-induced activation of excessive autophagy.

## Materials and Methods

### Cell Culture

The House Ear Institute-Organ of Corti 1 (HEI-OC1) cell line (Sigma-Aldrich, St. Louis, MO, United States) is a widely used progenitor HC line derived from the auditory organ of the transgenic mouse Immortomouse^TM^ that expresses molecular markers of cochlear cells (Kalinec et al., 2003; Jeong et al., 2005). The cell line was developed as an *in vitro* system to investigate the cellular and molecular mechanisms involved in ototoxicity and for screening the potential ototoxicity or otoprotective properties of pharmacological agents.

HEI-OC1 cells were grown under permissive conditions (33°C, 10% CO_2_) in high-glucose Dulbecco’s Modified Eagle’s Medium (DMEM; Gibco BRL, Gaithersburg, MD, United States) containing 10% fetal bovine serum (FBS; Gibco BRL) without antibiotics. All experiments concerning this cell line were conducted in the logarithmic growth phase.

### Drugs and Reagents

Cisplatin was from Hansoh Pharma, Jiangsu, China (Cat# 160203); sodium meclofenamate hydrate (MA) was from TCI, Japan (Cat# m1269); and compound MA2, the ethyl ester derivative of MA, was a gift from Professor CaiGuang Yang (CAS Key Laboratory of Receptor Research, Shanghai Institute of Materia Medica, Chinese Academy of Sciences, Shanghai, China) and was used to achieve better cell penetration. MA2 was diluted in dimethyl sulfoxide (DMSO, Solarbio, Beijing, China, Cat# D8370) to a stock concentration of 60 mM. Ly294002 (Cat# S1105), adenosine (Cat# S1647), and N^6^-methyladenosine (m^6^A) (Cat# S3190) were all from Selleckchem.com. Nuclease P1 from *Penicillium citrinum* (Cat# P8630), alkaline phosphatase (Cat# P7923), ammonium bicarbonate (Cat# V900254), and ammonium acetate (Cat# A1542) were all from Sigma-Aldrich.

### Cell Counting Kit-8 (CCK-8) for the HEI-OC1 Cell Viability Assessment

HEI-OC1 cells (5,000 cells/well) were seeded in 96-well flat-bottom plates (Corning Glass Works, Corning, NY, United States) in three replicates and incubated overnight under permissive conditions. After drug treatment in 100 μl culture medium, 10 μl CCK-8 (Biosharp, Shanghai, China) was added for 1.5 h. The optical density (OD) values were measured at 450 nm by an ELISA reader (Multiskan MK3, Shanghai Bio-excellent, Shanghai, China). The positive control underwent the same procedure, but without cell-seeding, whereas the negative control was just treated without drugs. The relative viability was calculated as: (OD experiment - OD positive)/(OD negative - OD positive) × 100.

### Protein Extraction and Western-Blot Analysis

Total protein from HEI-OC1 cells was extracted using RIPA Lysis Buffer (Beyotime Biotechnology, China), and the BCA Protein Quantification Kit (Beyotime Biotechnology) was used to measure the protein concentrations according to the manufacturer’s instructions. A total of 30 μg protein was denatured at 95°C and separated by 10% SDS-PAGE. The separated proteins were transferred to polyvinylidene fluoride membranes (PVDF, Immobilon-P, Cat# IPVH00010), and the membranes were blocked in TBS containing 0.1% Tween-20 (TBST) with 5% BSA and incubated with primary antibodies overnight at 4°C. After washing with TBST, the membranes were incubated with secondary antibodies, and the protein signal was detected using the chemiluminescence solutions in the ECL kit (Millipore, United States). The intensity of the protein bands was measured and analyzed using ImageJ software (Broken Symmetry Software, United States). β-actin was used as the loading control. The primary antibodies were anti-LC3-II (#3868, Cell Signaling Technology, United States), anti-caspase3 (#9665, Cell Signaling Technology, United States), and anti-β-actin (sc-1615 HRP, Santa Cruz Biotechnology, United States).

### Flow Cytometry Assay of Apoptosis

The rate of apoptosis in HEI-OC1 cells was quantitatively determined with Annexin V-fluorescein isothiocyanate (FITC)/propidium iodide (PI) (Sigma-Aldrich) double staining and flow cytometry.

Cells were seeded in six-well culture plates with 80 μM MA2 for 2 h and then treated with 15 μM cisplatin for 48 h. Culture medium with vehicle alone was used as the control. After collection, cells were washed with PBS and resuspended in 500 μl 1× binding buffer. Cells were transferred into fluorescence-activated cell sorting tubes and stained using the Annexin V-FITC apoptosis detection kit following the manufacturer’s protocols (Sigma-Aldrich). The specific binding of Annexin V-FITC occurred during incubation of the cells for 15 min at room temperature in binding buffer containing saturating concentrations of Annexin V-FITC and PI. Afterward, the cells were analyzed by flow cytometry (BD Biosciences, Heidelberg, Germany) for a cell count of 20,000. The cells were analyzed by flow cytometry as soon as possible, and all experiments were repeated at least three times.

### Measurement of Intracellular ROS Production

The intracellular ROS level was measured with the CellROX Deep Red Reagent (Molecular Probes, Life Technologies, United States). For the assay, HEI-OC1 cells were cultured overnight in six-well plates and then were treated as described above. HEI-OC1 cells were incubated in the dark for 30 min at 37°C with 5 μM CellROX Deep Red Reagent. Fluorescence was analyzed by using a FACSCalibur flow cytometer (BD Biosciences, United States) at an excitation wavelength of 635 nm (FL-4) with gating at 20,000 cells/sample.

### Transmission Electron Microscopy (TEM)

HEI-OC1 cells were collected by trypsinization and were immediately fixed with 3% glutaraldehyde fixative solution (pH 7.4) for 1 h followed by 1% osmic acid (OsO_4_) in 0.1 M sodium cacodylate buffer (pH 7.2) for 1–2 h. The cells were then dehydrated with acetone and embedded in araldite CY212. Ultrathin sections (70 nM) were cut with a diamond knife, mounted on EM grids, stained with Reynolds’ lead citrate solution, gently washed with distilled water, dried, and imaged using a JEM-1200 EX electron microscope (FEI, Hillsboro, OR, United States).

### LC3B Immunofluorescence Staining

HEI-OC1 cells were cultured in 48-well dishes with DMEM culture medium plus 10% FBS. At the end of each experimental treatment, we aspirated the liquid and covered the cells to a depth of 2–3 mm with ice-cold 100% methanol. After fixing for 15 min at -20°C, we aspirated the fixative, rinsed three times in 1× PBS for 5 min each time, and then performed the immunostaining according to the manufacturer’s protocol. Briefly, specimens were blocked in blocking buffer for 1 h and then incubated overnight with primary antibody (#3868, Cell Signaling Technology, United States. 1:200 dilution) at 4°C. Samples were then treated with secondary antibodies and diamidino-phenyl-indole (DAPI, 1:1,000 dilution) for 1 h and visualized with a confocal microscope (Leica, Wetzlar, Germany). Negative controls without primary antibodies were performed to test the specificity of the antibodies.

### mRNA Isolation and Quantitative Analysis of m^6^A

The total RNA was extracted using Trizol Reagent (Invitrogen, Carlsbad, CA, United States) according to the manufacturer’s protocol. The mRNA was isolated using a Dynabeads mRNA Purification Kit (for mRNA purification from total RNA) (Thermo-Fisher, Cat# 61006) according to the manufacturer’s protocol. It is important to point out that after acquiring the isolated mRNA from the total RNA, we once again washed the used Dynabeads in 1× binding buffer and performed another capture of mRNA from the above product following the same process to acquire the purified mRNA. The concentration of mRNA was measured by NanoDrop, and an Agilent 2100 Bioanalyzer was used to analyze the RNA quality with an RNA NanoChip.

A total of 0.5 μg of mRNA was digested by nuclease P1 (1 Unit) in 40 μl of buffer containing 10 mM NH_4_Ac (pH 5.3) at 42°C for 4 h, followed by the addition of NH_4_HCO_3_ (1.0 M, 3 μl) and alkaline phosphatase (0.5 Unit). The solution was diluted 5–20 fold, and 5 μl of the solution was injected into the LC-MS/MS system after an additional incubation at 37°C for 3 h.

Reverse-phase high-performance liquid chromatography on a C18 column was used to separate the nucleosides. An Agilent 6460 QQQ triple-quadrupole LC mass spectrometer in positive electrospray ionization mode was used to detect the mass spectrometry online.

The nucleosides were quantified using the nucleoside to base ion mass transitions of 282 to 150 (m^6^A) and 268 to 136 (A). Quantification was performed by comparison with the standard curve obtained from pure nucleoside standards running on the same batch of samples. The ratio of m^6^A to A was determined based on the calculated concentrations.

### Statistical Analysis

All statistical analyses were performed in the SPSS v17 software package. One-way ANOVA was used to compare groups, and all data were expressed as the mean ± SD. A *p*-value of less than 0.05 was considered statistically significant.

## Results

### MA2 Protects Against Cisplatin-Induced Cytotoxicity in HEI-OC1 Cells

**FIGURE 1:**
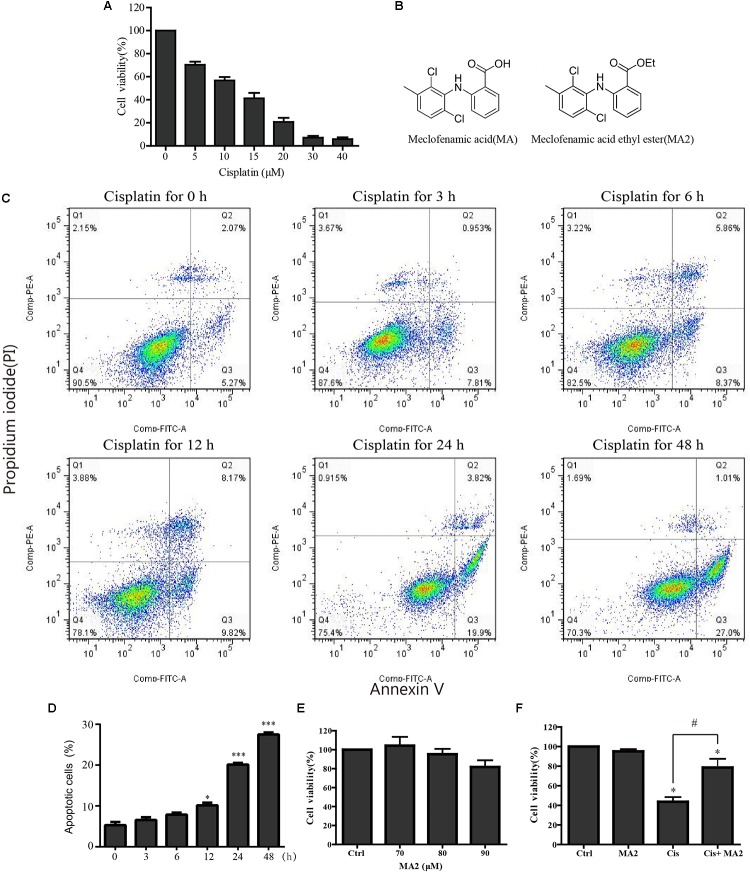
**(A)** HEI-OC1 cells were incubated with different concentrations of cisplatin (from 0 to 40 μM) for 48 h, and cell viability was measured with the CCK-8 kit. **(B)** The chemical structure of MA and MA2. **(C)** Flow cytometry was used to measure apoptosis after 15 μM cisplatin treatments for different times. **(D)** Quantification of the data in **(C)**. **(E)** After treatment with different concentrations of MA2 (0, 70, 80, and 90 μM) for 48 h, the cell viability was measured with the CCK-8 kit. **(F)** The cells were incubated with 15 μM cisplatin in the presence or absence of 80 μM MA2 for 48 h, and the cell viability was measured with the CCK-8 kit. ^∗^*p* < 0.05 compared to the DMSO-only group. ^#^*p* < 0.05 comparing the cisplatin-only group and the cisplatin + MA2 group, determined using one-way ANOVA.

In this study, HEI-OC1 cells were used to investigate the protective effect of MA2 on cisplatin-induced cytotoxicity. First, to determine the optimum cisplatin concentration for inducing HEI-OC1 cell damage, the cells were treated with different cisplatin concentrations (0, 5, 10, 15, 20, 30, and 40 μM) for 48 h. The CCK-8 values, which show the cell viability, were 100%, 70.3 ± 2.69%, 56.79 ± 2.97%, 41.21 ± 4.71%, 20.72 ± 3.67%, 7.14 ± 1.48%, and 5.88 ± 1.42% for each cisplatin concentration, respectively (**Figure 1A**). We measured the apoptosis levels after 15 μM cisplatin treatment for different times (0, 3, 6, 12, 24, and 48 h), and the percentage of apoptotic cells increased with the time of cisplatin treatment (5.29 ± 0.77%, 6.55 ± 0.65%, 7.85 ± 0.52%, 10.13 ± 0.65%, 20.1 ± 0.43%, and 27.47 ± 0.5%, respectively) (**Figures 1C,D**). We chose 15 μM cisplatin treatment for 48 h as an appropriate condition for HEI-OC1 cells injury (the number of viable cells was decreased to less than 50% compared to the control). Thus, we used this cisplatin concentration for all of the experiments in this study. We next determined the optimum concentration of MA2 (the molecular structures of MA and MA2 are shown in **Figure 1B**). The cells were treated with different MA2 concentrations (0, 70, 80, and 90 μM) for 48 h, and the CCK-8 assay showed cell viabilities of 100%, 104.26 ± 9.33%, 95.22 ± 5.67%, and 81.73 ± 7.08%, respectively (**Figure 1E**). Thus, we used 80 μM MA2, which was the highest dose showing no significant cell death, for the following experiments.

To further examine the cytoprotective effects of MA2 in cisplatin injury, we divided the experimental samples into the following four groups: the DMSO-only group, the MA2-only group, the cisplatin-only group, and the cisplatin + MA2 group. The cell viability results showed that the proportions of viable cells in the cisplatin-only group (43.67 ± 4.74% viable cells) and the cisplatin + MA2 group (78.66 ± 8.89% viable cells) were significantly decreased compared to the control group (100% viable cells) (**Figure 1F**). Moreover, we found that the cell viability was significantly enhanced by MA2 after cisplatin injury. Together, these results suggest that MA2 plays a protective role in cisplatin-induced cytotoxicity in HEI-OC1 cells.

### MA2 Attenuates Cisplatin-Induced HEI-OC1 Cell Apoptosis

**FIGURE 2:**
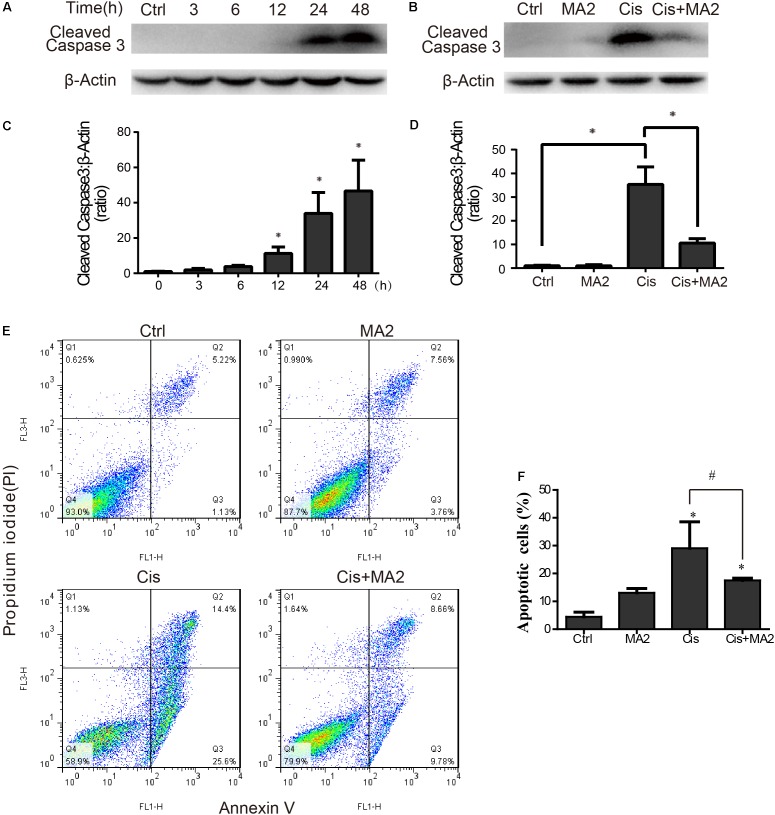
**(A)** HEI-OC1 cells were incubated with 15 μM cisplatin for 0, 3, 6, 12, 24, or 48 h, and then western blots were performed with anti-cleaved-caspase3 antibody. **(B)** HEI-OC1 cells were incubated with 15 μM cisplatin for 48 h with or without 80 μM MA2, and then western blots were performed with anti-cleaved-caspase3 antibody. **(C)** Quantification of the western blot in **(A)**. **(D)** Quantification of the western blot in **(B)**. **(E)** Flow cytometry was used to measure the rate of apoptosis after different treatments. HEI-OC1 cells were incubated with 15 μM cisplatin for 48 h with or without 80 μM MA2 and then stained with Annexin V-FITC/PI. Cells were analyzed by flow cytometry for a cell count of 20,000, and each experiment was repeated at least three times. In the flow cytometry plots, the Annexin V-negative and PI-negative quadrant (lower left quadrant, Q4) defines viable cells, the Annexin V-positive and PI-negative quadrant (lower right quadrant, Q3) defines early apoptotic cells, and the Annexin V-positive and PI-positive quadrant (upper right quadrant, Q2) defines late apoptotic cells and necrotic cells. **(F)** Quantification of the data in **(E)**. The proportion of apoptotic cells (Q2 + Q3) increased significantly after cisplatin treatment. Data are shown as the mean ± SEM of three replicates, ^∗^*p* < 0.05 compared with the control. ^#^*p* < 0.05 comparing the cisplatin-only group to the cisplatin + MA2 group using one-way ANOVA.

Western blot showed that the protein expression of cleaved-caspase3 (the activated form of caspase-3) was significantly increased after cisplatin treatment in HEI-OC1 cells compared to the undamaged controls as the exposure time increased and that it reached its highest level at 48 h (**Figures 2A,C**). The protein expression level of cleaved-caspase3 was significantly reduced after 80 μM MA2 treatment compared to the cisplatin-only group (**Figures 2B,D**). Next, we performed flow cytometry experiments. The dead cells were labeled by PI and the cells undergoing apoptosis were labeled by Annexin V. The proportion of apoptotic cells was significantly increased after cisplatin treatment (29.05 ± 9.50% apoptotic cells) compared to the undamaged control (4.44 ± 1.68% apoptotic cells) (**Figures 2E,F**). We also found that the proportion of apoptotic cells (17.51 ± 0.81% apoptotic cells) was significantly reduced after treatment with MA2 compared to the cisplatin-only group (**Figures 2E,F**). Taken together, these results suggest that MA2 significantly reduces cisplatin-induced HEI-OC1 cell apoptosis.

### MA2 Attenuates Cisplatin-Induced Oxidative Stress in HEI-OC1 Cells

**FIGURE 3:**
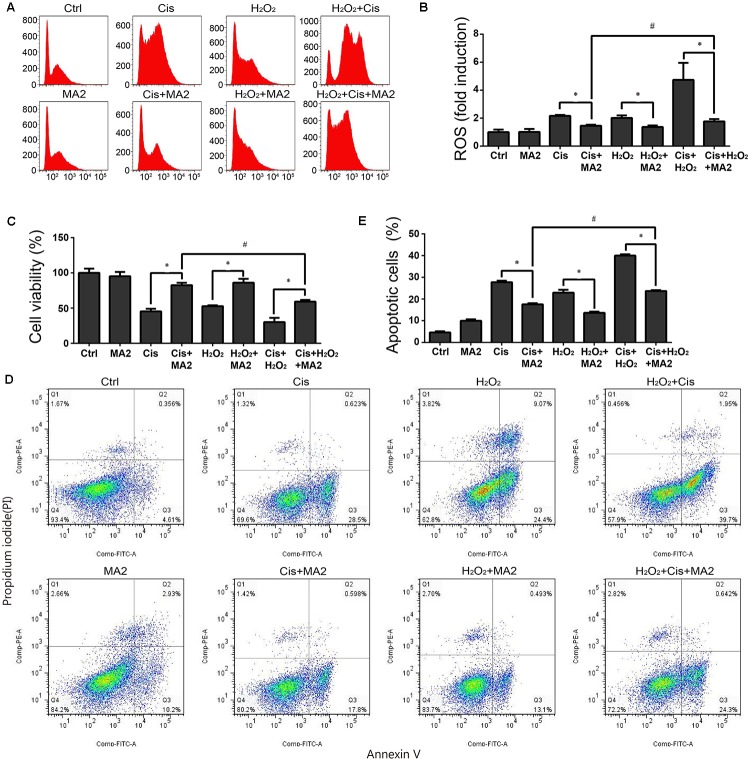
**(A)** HEI-OC1 cells were incubated with 15 μM cisplatin and H_2_O_2_ for 48 h with or without 80 μM MA2 and then stained with CellROX^®^ Deep Red. Cells were analyzed by flow cytometry for a cell count of 20,000, and all experiments were repeated at least three times. The levels of intracellular ROS were increased significantly after cisplatin treatment, and MA2 significantly inhibited the production of intracellular ROS. **(B)** Quantification of the data in **(A)**. **(C)** The cells were incubated with different treatments, and the cell viability was measured with the CCK-8 kit. **(D)** Flow cytometry was used to measure apoptosis after different treatments. **(E)** Quantification of the data in **(D)**. Data are presented as the means ± SD of triplicate determinations from three independent experiments. ^∗^*p* < 0.05 compared with the control. ^#^*p* < 0.05 comparing the cisplatin + MA2 group to the cisplatin + H_2_O_2_+MA2 group using one-way ANOVA.

ROS have been reported have a close relationship with the process of ototoxic drug-induced HC damage (Choung et al., 2009; Chen et al., 2015). To measure changes in mitochondrial ROS in cisplatin-treated HEI-OC1 cells after MA2 treatment, we used CellROX^®^ Deep Red, which is a redox fluorophore that selectively measures cellular oxidative stress (He et al., 2017). The flow cytometry results showed that the ROS levels were increased 2.16 ± 0.07-fold after cisplatin treatment compared to the undamaged controls (**Figures 3A,B**) and that the levels of ROS were markedly reduced by MA2 compared with the cisplatin-only group (fold changes of 1.45 ± 0.07 and 2.16 ± 0.07, respectively) (**Figures 3A,B**). In addition, the ROS levels were unchanged in the MA2-only group (1.01 ± 0.21 fold change). In addition, we used H_2_O_2_ to activate ROS production in HEI-OC1 cells. The results showed that the ROS levels were increased 2.01 ± 0.18-fold after H_2_O_2_ treatment compared to the undamaged controls (**Figures 3A,B**) and that the levels of ROS were markedly reduced by MA2 treatment compared with the H_2_O_2_-only group (fold changes of 1.37 ± 0.1 and 2.01 ± 0.18, respectively) (**Figures 3A,B**). We also found that the ROS levels were decreased after cisplatin, MA2, and H_2_O_2_ co-treatment compared to cisplatin and H_2_O_2_ treatment (fold changes of 1.77 ± 0.16 and 4.73 ± 1.21, respectively) (**Figures 3A,B**). These results demonstrated that MA2 could attenuate the ROS levels in HEI-OC1 cells after cisplatin exposure.

When we measured the cytoprotective effects of MA2 together with H_2_O_2_ treatment, the cell viability results showed that the cell numbers in the cisplatin, MA2, and H_2_O_2_ co-treatment group (59.22 ± 2.29% viable cells, controls were considered as 100%) were significantly decreased compared to the cisplatin + MA2 group (80.54 ± 3.44% viable cells) (**Figure 3C**). Next, the flow cytometry experiments showed that the Annexin V-positive apoptotic cells were significantly increased in the cisplatin, MA2, and H_2_O_2_ co-treatment group (24.7 ± 1.4% apoptotic cells) compared to the cisplatin + MA2 group (17.56 ± 0.49% apoptotic cells) (**Figures 3D,E**). Together, these results demonstrated that MA2 reduces apoptosis by inhibiting the increase in intracellular ROS levels in HEI-OC1 cells after cisplatin exposure.

### Autophagy Increases in HEI-OC1 Cells After Cisplatin Exposure

**FIGURE 4:**
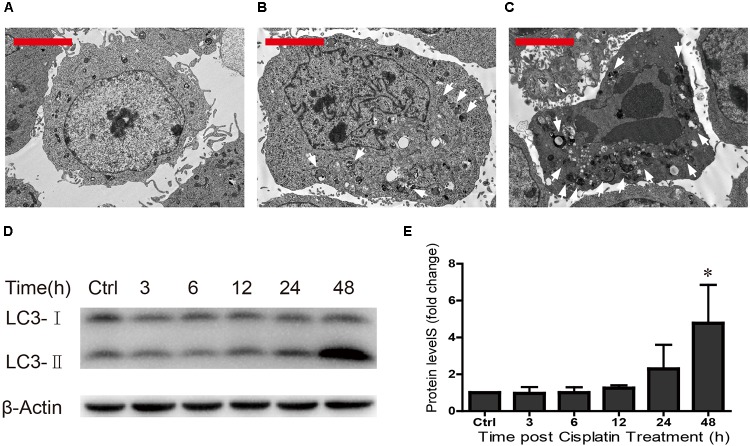
TEM was used to determine cell morphology in **(A)** the undamaged HEI-OC1 cells, **(B)** after cisplatin treatment for 24 h, and **(C)** after cisplatin treatment for 48 h (arrows in the pictures indicate the autophagic lysosomes). **(D)** Western blot results showing LC3-II expression changes after different exposure times (0, 3, 6, 12, 24, and 48 h). β-actin served as the sample loading control. **(E)** Quantification of the western blot in d. ^∗^*p* < 0.05 compared with the control. *n* = 3. Scale bars = 2 μm.

Previous studies have shown that ototoxic drug-induced HC loss is commonly associated with the accumulation of ROS and the induction of autophagy, which is a cellular defense pathway (Marcotti et al., 2005; Mcfarland et al., 2007; Vernon and Tang, 2013; Yuan et al., 2015). Because autophagy plays an important role in cell survival and because excessive autophagy activation induces cell death and pathological changes (Clarke, 1990; Yu and Lenardo, 2004; Levine and Kroemer, 2008; Meijer and Codogno, 2009; Czaja et al., 2013; Ryter et al., 2014), we measured the levels of autophagy after different cisplatin exposure times (0, 24, and 48 h) in HEI-OC1 cells. TEM was used to determine the morphology of the cells and the occurrence of autophagy. We found that in undamaged cells the cytoplasmic membrane was intact, the microvilli were clearly visible, the mitochondria exhibited an oval or round shape with almost no cavitation, and the nuclei were round and clear (**Figure 4A**). After cisplatin exposure for 24 h, the plasma membrane was still intact, the microvilli were still visible, the mitochondria were almost normal but showed a small amount of mitochondrial cavitation, a small number of autophagic lysosomes were present in the cytoplasm, and nuclear atypia was obvious (**Figure 4B**). After cisplatin exposure for 48 h, chrysanthemum-like bodies were present throughout the cells, a large number of autophagic lysosomes appeared in the cytoplasm, there was nuclear and cytoplasmic condensation, the electron density increased, and there was significant heterochromatin aggregation (**Figure 4C**).

The Western blot results revealed that LC3-II expression increased as the cisplatin exposure time increased (fold changes in LC3-II levels were 1, 0.96 ± 0.35, 1.00 ± 0.30, 1.25 ± 0.15, 2.29 ± 1.31, and 4.77 ± 2.09, respectively) (**Figures 4D,E**). We found that cisplatin exposure for 48 h result in the highest level of LC3-II. These results suggested that the cisplatin-induced excessive autophagy might be involved in HEI-OC1 apoptosis.

### MA2 Treatment Inhibits the Cisplatin-Induced Activation of Excessive Autophagy

**FIGURE 5:**
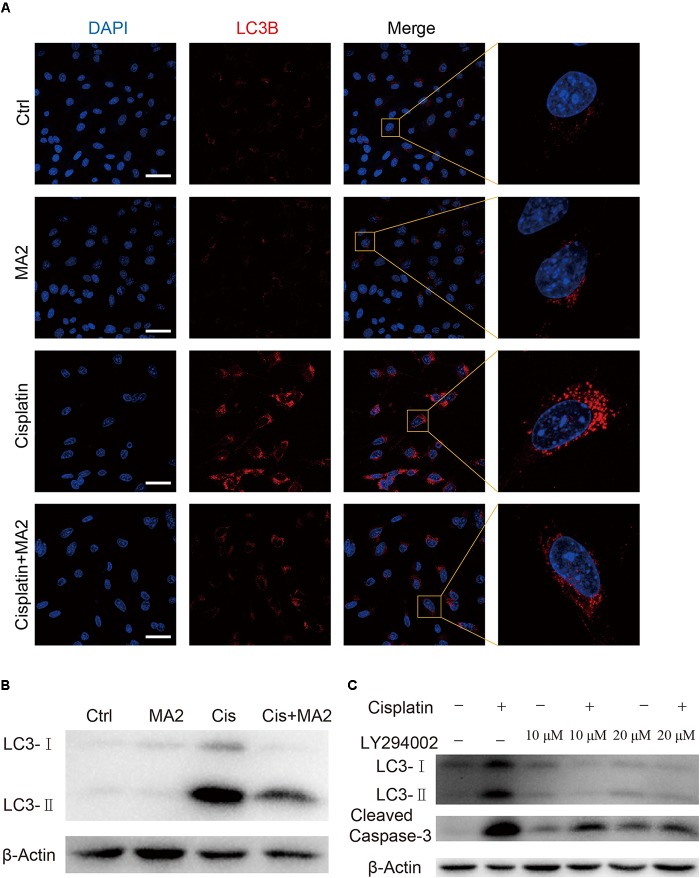
**(A)** Immunofluorescence staining with LC3B (red) in HEI-OC1 cells after 15 μM cisplatin treatment for 48 h with or without 80 μM MA2. **(B)** Western blotting showed changes of LC3-II expression after cisplatin and MA2 treatment. GAPDH served as the sample loading control. **(C)** Western blotting showed changes of LC3 and cleaved caspase-3 expression after 15 μM cisplatin treatment for 48 h with or without 10 or 20 μM LY294002. Scale bars = 50 μm.

In this study, we sought to determine the role of MA2 in autophagy regulation in HEI-OC1 cells after cisplatin exposure. To determine whether LC3 expression was affected by MA2, immunofluorescence staining with anti-LC3B antibodies was performed. Treatment with cisplatin resulted in increased autophagosome formation in the cytoplasm of HEI-OC1 cells, and co-treatment with MA2 and cisplatin markedly reduced the autophagosome formation in the cytoplasm compared to the cisplatin-only group (**Figure 5A**). The western blots also showed that cisplatin significantly stimulated the expression of LC3 and that this trend was reduced by MA2 (**Figure 5B**).

To determine whether cisplatin-induced activation of autophagy plays a role in apoptosis after cisplatin treatment, we treated the cells with 10 or 20 μM of the autophagy inhibitor LY294002, which inhibits PI3K/Akt signaling, with or without cisplatin. The western blots showed that LY294002 treatment decreased both autophagosome formation and apoptosis (**Figure 5C**), suggesting that excessive activation of autophagy is also involved in apoptosis after cisplatin treatment. We conclude, therefore, that MA2 treatment inhibited the cisplatin-induced activation of autophagy, which in turn inhibited apoptosis after cisplatin exposure.

### MA2 Does Not Inhibit Demethylases After Cisplatin Exposure

**FIGURE 6:**
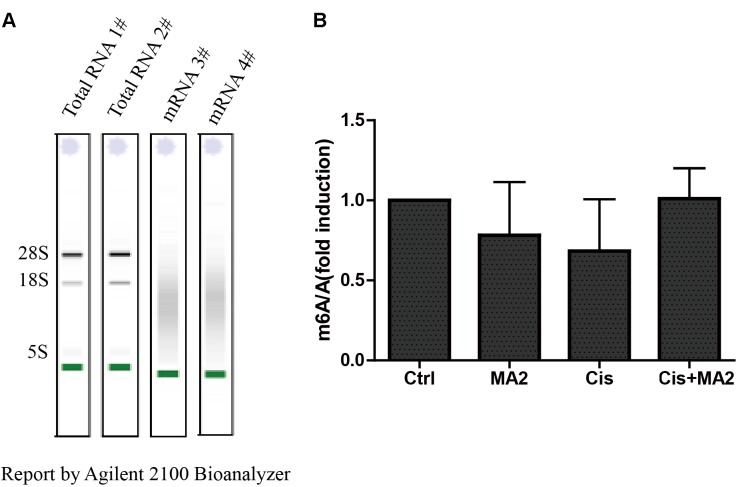
**(A)** The quality of mRNA analyzed by an Agilent 2100 Bioanalyzer. **(B)** The m^6^A/A ratio in the four groups of samples normalized to the DMSO control group. There were no significant differences in the m^6^A/A ratio in any of the groups.

MA is a highly selective inhibitor of FTO by competing with FTO for binding to m^6^A-containing nucleic acids, and its ethyl ester form (MA2) can increase the levels of m^6^A modifications in mRNA in HeLa cells (Huang et al., 2015). To further verify whether MA2 acts as an inhibitor of demethylases in HEI-OC1 cells, we analyzed the m^6^A/A ratio after 80 μM MA2 treatment. First, we isolated the mRNA and analyzed the quality of the mRNA on an Agilent 2100 Bioanalyzer with an RNA NanoChip. The results showed that both the mRNA purity and quality met our experimental requirements (**Figure 6A**). The mRNA was then hydrolyzed into single nucleosides and analyzed on a mass spectrometer for the determination of the content of m^6^A and A. We found that there was no significant increase in the m^6^A/A ratio after MA2 treatment (**Figure 6B**). The m^6^A/A ratio in the DMSO group, MA2-only group, cisplatin-only group, and cisplatin+MA2 group was 1, 0.78 ± 0.33, 0.68 ± 0.32, and 1.01 ± 0.19, respectively. Together, these results show that the inhibition of cisplatin-induced apoptosis in HEI-OC1 cells by MA2 might not be by directly targeting the level of m^6^A.

## Discussion

Hearing impairment is the most prevalent sensorial deficit in the general population, and it is caused by different etiologies such as congenital morphogenetic defects, aging, exposure to intense noise, ototoxic medications, and genetic disorders (Fujimoto et al., 2017; Mammano and Bortolozzi, 2017). HCs in the inner ear are specialized sensory cells that play an essential role in converting mechanical sound movement to neural signals for hearing (Chai et al., 2012). One of the main causes of sensorineural hearing loss is the HC damage induced by ototoxic drugs, and the major classes of ototoxic drugs are loop diuretics, aminoglycosides, and chemotherapeutics (He et al., 2017). Cisplatin is one of the most widely used chemotherapeutic agents for the treatment of neoplastic diseases such as ovarian cancer, lung cancer, head and neck cancer, testicular cancer, bladder cancer, and cervical cancer, but the ototoxicity of cisplatin restricts its utility and clinical applications (Rybak et al., 2007). Thus finding a way to attenuate cisplatin-induced HC loss is a primary focus in hearing research. Because RNA epigenetic modifications and autophagy are important for many aspects of biology, such as cellular signaling, cell development, cell cycle regulation, and apoptosis, we sought to demonstrate the role of RNA epigenetic modifications and autophagy in the process of cisplatin-induced HC loss.

RNA plays a central role in the transfer of biological information, and it plays roles in maintaining the status and functions of cells through interactions with proteins and DNA. Thus, methylation and other epigenetic modifications of RNA play a key role in regulating RNA function, and deficiencies in epigenetic modifications have been shown to be behind many progressive neurodegenerative conditions such as Huntington’s disease (Taylor and Fischbeck, 2002). In the inner ear, epigenetic modifications might also be related to inner ear development and have a significant role in hearing loss, hearing protection, and regeneration of functional cells. The mRNA modifications begin at the 5′ end of the transcript, including 2′-*O*-methylated ribonucleotides such as *N*^6^,2′-*O*-dimethyladenosine (m^6^Am), *N*^6^,*N*^6^,2′-*O*-trimethyladenosine (m^6^_2_Am), and 3,2′-*O*-dimethyluridine (m^3^Um), which define the beginning of the transcripts (Bangs et al., 1992; Tschudi and Ullut, 2002). The m^6^A modification has important roles in regulating crucial cellular pathways and processes in a wide range of eukaryotes (Rottman et al., 1977), and dysregulation of this modification might contribute to obesity, brain development abnormalities, and other diseases (Klungland and Dahl, 2014; Geula et al., 2015).

RNA demethylation regulates the expression of downstream RNA-processing genes and epigenetic modifying genes. FTO and ALKBH5^8^ are two m^6^A demethylases that control mRNA metabolism by catalyzing the demethylation of m^6^A (Wang et al., 2014). FTO mediates the oxidative demethylation of nucleotide bases, and the knockdown of FTO leads to increased amounts of m^6^A in mRNA, whereas overexpression of FTO results in decreased amounts of m^6^A in human cells (Jia et al., 2011). The non-steroidal, anti-inflammatory drug MA acts as a highly selective inhibitor of FTO by competing with FTO for binding to m^6^A-containing nucleic acids (Huang et al., 2015). In this study, we tested the levels of apoptosis in HEI-OC1 cells induced by cisplatin when FTO activity was inhibited by MA2, which is the more active ethyl ester form of MA (Huang et al., 2015). We found that the number of apoptotic HEI-OC1 cells after cisplatin exposure was significantly reduced by MA2 (**Figures 1**, **2**). Due to the ability of MA2 to elevate levels of cellular m^6^A in mRNA by targeting FTO, we asked whether MA2 reduces cisplatin-induced HEI-OC1 cell apoptosis by regulating the level of m^6^A in total mRNA. We found that there was no significant increase in the level of m^6^A after MA2 treatment (**Figure 6**), thus we speculate that MA2’s effects on the process of cisplatin-induced HEI-OC1 apoptosis might not be by directly targeting the level of m6A.

Several previous studies have shown that HC loss induced by ototoxic drugs is commonly associated with ROS accumulation, which induces mitochondrial depolarization and initiates apoptosis (Marcotti et al., 2005; Mcfarland et al., 2007; Shan et al., 2014; Guan et al., 2016; He et al., 2016; Liu et al., 2016; Yu et al., 2017). We evaluated the levels of mitochondrial ROS in HEI-OC1 cells after cisplatin and H_2_O_2_ treatment and found that the ROS level was significantly decreased after MA2 treatment compared to the cisplatin and H_2_O_2_ treatment group (**Figure 3**). ROS can induce cellular defense pathways, including autophagy, which can recycle unnecessary or dysfunctional cellular components (Vernon and Tang, 2013; Yuan et al., 2015). Autophagy is important for cell survival, and dysregulation of autophagy is the cause of many diseases, and excessive autophagy activation can induce cell death and pathological changes through overactive degradative processes (Clarke, 1990; Yu and Lenardo, 2004; Levine and Kroemer, 2008; Meijer and Codogno, 2009; Czaja et al., 2013; Ryter et al., 2014). Autophagy, as another kind of programmed cell death, is a series of biochemical processes in which eukaryotic cells perform “self-digestion” by degrading their own cytoplasm and organelles. Autophagy promotes cell death through coordinated transformation with apoptosis (Baehrecke, 2005). By measuring the autophagic flux via the expression of LC3-II, which is an autophagy marker, and by TEM imaging, we found that autophagy was significantly increased after exposure to 15 μM cisplatin for 48 h (**Figures 4**, **5**) and that the overactivation of autophagy was significantly inhibited when HEI-OC1 cells were treated with MA2 or with the autophagy inhibitor LY294002. Moreover, we found that the autophagy inhibitor LY294002 can also decrease cisplatin-induced cell apoptosis (**Figure 5**), suggesting that excessive activation of autophagy also takes part in cisplatin-induced apoptosis. However, autophagy was only significantly activated 48 h after cisplatin treatment, while there was already a significant amount of apoptosis at 24 h; thus the autophagy inhibitor LY294002 only partially decreased the level of apoptosis. These results suggest that excessive autophagy plays a role in cisplatin-induced apoptosis, but is not the main cause of cisplatin-induced apoptosis. In the process of apoptosis caused by cisplatin treatment, excessive autophagy might be one of the factors that promotes apoptosis, but not the main factor leading to apoptosis. Together, these results indicate that MA2 reduces cisplatin-induced apoptosis and inhibits excessive autophagy.

In summary, this study shows that cisplatin exposure causes ROS accumulation and excessive autophagy activation in HEI-OC1 cells and that MA2 plays an important role in HEI-OC1 cell survival after cisplatin exposure. Our results suggest that MA2 can inhibit apoptosis and prevent the overactivation of autophagy after exposure to cisplatin and that it does so not by directly regulating the level of m^6^A. Our findings provide new insights into new chemical probes for studies on the roles of epigenetics in ototoxicity and suggest potential therapeutic targets for the amelioration of cisplatin-induced ototoxicity.

## Author Contributions

RC and ZF conceived and designed the experiments. HL, YS, XW, XL, XB, and WL performed the experiments. BL performed the TEM experiment. SW performed the LC-MS/MS experiment. HL, ZH, and XC wrote the paper. YH, LX, DZ, JL, and HW helped interpret the data and wrote the paper.

## Conflict of Interest Statement

The authors declare that the research was conducted in the absence of any commercial or financial relationships that could be construed as a potential conflict of interest. The reviewer ZW declared a shared affiliation, with no collaboration, with several of the authors, HL, XC, and XW, to the handling Editor.
